# Physical Exercise and Undergraduate Students’ Subjective Well-Being: Mediating Roles of Basic Psychological Need Satisfaction and Sleep Quality

**DOI:** 10.3390/bs12090316

**Published:** 2022-08-30

**Authors:** Sen Lin, Liming Li, Dong Zheng, Libiao Jiang

**Affiliations:** 1 School of Humanities and Social Sciences, Xi’an Jiaotong University, Xi’an 710049, China; 2 Department of Physical Education, Shaanxi University of Science & Technology, Xi’an 710021, China

**Keywords:** physical exercise, well-being, basic psychological need satisfaction, sleep quality, self-determination theory

## Abstract

This study aimed to examine the association between physical exercise and subjective well-being among undergraduate students, as well as its underlying mechanism—the potential mediating roles of basic psychological need satisfaction and sleep quality—from the perspective of self-determination theory. A sample of 770 undergraduate students (mean age was 19.90 ± 1.15 years old; 464 women) were recruited voluntarily to complete a set of measures examining physical exercise habits, psychological need satisfaction in exercise, sleep quality, and subjective well-being. The results indicated that (1) physical exercise was positively associated with undergraduate students’ subjective well-being; (2) psychological need satisfaction and sleep quality could significantly mediate this relation, which contained three pathways—the independent mediating effects of sleep quality and basic psychological need satisfaction and the sequential mediating effect of them. These findings not only reveal the mediating mechanism underlying the relation between physical exercise and subjective well-being by integrating the psychological and physical factors together but also provide an empirical basis for formulating prevention and intervention programs aimed at promoting the health and subjective well-being of undergraduate students.

## 1. Introduction

Amid economic and societal development, people are paying increasing attention to healthy lifestyles and well-being. Regular physical exercise is a core element and indicator of a healthy lifestyle, which can bring significant physical and mental benefits to individuals. However, current studies tend to focus on unhealthy groups (e.g., individuals with chronic disease) and examine the association between physical exercise and negative indicators, such as depression, anxiety, or suicide [1,2]. In particular, along with the conveniences brought by modern information technology and the acceleration of the pace of modern life, the lack of time to participate in physical exercise, sedentary behavior, and screen use have correspondingly become increasingly common, and these are risk factors for individuals’ health and adaptation—especially among young teenagers [3,4]. Therefore, physical exercise and its benefits deserve more attention due to their theoretical and practical significance.

In addition, with the popularity of positive psychology, increasing attention has been paid to the positive psychological traits and affect, such as subjective well-being. Subjective well-being refers to the general joy and pleasurable emotions based on life satisfaction, which is an important indicator of individuals’ good adaptation and is closely associated with numerous indicators of adaptation [5,6]. Against this background, the influencing factors accounting for subjective well-being have become the focus of research and society, among which physical exercise is getting more and more attention. Though the relation between physical exercise and adaptation (including well-being) has been examined, the underlying mechanism is largely unclear and should be further examined.

In particular, undergraduate students are in a critical transitional period, facing a variety of pressures and challenges, as well as less external control and supervision when compared to their previous stages of life; they are thus more likely to suffer from psychological problems and generally have a low level of well-being [7]. Meanwhile, they are also extremely active in various Internet applications or screen usage while being inactive in physical exercise or sports (for example, sedentary behavior is common among these students) [3,8]. Based on this evidence, this study aimed to examine the association between physical exercise and subjective well-being among undergraduate students and the mechanisms underlying this relation.

### 1.1. Physical Exercise and Subjective Well-Being

Physical exercise refers to body movements made by skeletal muscles via the expenditure of energy. There are many health and adaptation benefits associated with physical exercise; specifically, regular physical exercise can promote both physical health (e.g., physiological function, such as vital capacity and strength, ideal body fat percentage, and body mass index) and psychological adaptation [9,10]. Several studies have shown that physical exercise has a negative relationship with perceived stress, depression, anxiety, suicide attempts, and even suicidal behavior but a positive relationship with self-esteem, self-efficacy, psychological capital, and life satisfaction [1,2,11]. From the opposite perspective, the lack of necessary physical exercise, as in sedentary behavior and excessive screen time, negatively influences individuals’ health and adaptation, being linked with obesity and overweight, depression, low quality of life, and low self-esteem [12,13,14]. Physical exercise interventions have been widely adopted to improve individuals’ physical and psychological functions; these improve physical functioning and sleep quality, promote positive emotions, and decrease perceived stress and anxiety [15,16]. Physical exercise may also be adopted to improve health conditions in stressful environments, such as the COVID-19 pandemic [17]. Thus, it was hypothesized that physical exercise is positively associated with undergraduate students’ subjective well-being based on the evidence.

### 1.2. Mediating Roles of Basic Psychological Need Satisfaction

Self-determination theory proposes that all human beings are motivated by three basic psychological needs—the need for competence (effectiveness or capacities to achieve desired outcomes), autonomy (freedom and self-direction in activities), and relatedness (meaningful connection with others)—the satisfactions of which are of essential significance for individuals’ health and adaptation [18]. Indeed, the satisfaction of these basic psychological needs should stimulate intrinsic motivation and contribute to positive life outcomes, such as less depression, anxiety, and problem behaviors (e.g., smoking, alcohol, or drug use); higher self-esteem and quality of life; and more positive emotions [19,20]. Regarding well-being, its close and positive association with satisfaction of basic psychological needs has been confirmed across diverse participants [21,22].

The extent to which basic needs are satisfied is usually derived from the environment and individuals’ experiences, such as life events, social contexts, interpersonal relationships, and daily activities [18]. Self-determination theory (especially its points of view on psychological needs) has been applied to the field of physical exercise, with the aim of expounding the motivation to engage in physical exercise and its outcomes [20,23,24]. Empirical studies have found that regular participation in physical exercise is a primary way to satisfy the basic needs for competence, autonomy, and relatedness, and that it can do so by providing a supportive environment that facilitates a sense of volition, optimal challenges, positive feedback, and a sense of belonging and acceptance. The satisfaction of these basic psychological needs is the key factor accounting for the positive influence of physical exercise on individuals’ health and adaptation [15,23,25]. Based on the theoretical and empirical evidence, it was hypothesized that basic psychological need satisfaction could mediate the association between physical exercise and well-being.

### 1.3. Mediating Roles of Sleep Quality

Sleep quality is another social issue attracting much attention that has been identified as a public health problem by the World Health Organization, and an increasing number of people report poor sleep quality [26]. Sleep quality is an important indicator of and is closely related to physical and psychological health. Specifically, good sleep quality promotes individuals’ health and well-being, whereas poor sleep quality results in various adverse outcomes, such as health problems (e.g., obesity, cardiovascular disease), impaired cognitive functioning, poor academic performance, perceived stress, psychological pain, depression, and even suicide [27,28,29]. Predictors of sleep quality have been widely examined. Recently, the relation between physical exercise and sleep quality has also attracted the attention of researchers. Research has indicated that participants regularly engaging in sports or physical exercise have better sleep patterns—fewer awakenings, less daytime sleepiness, better sleep duration, and higher sleep quality [12,30]. Thus, physical exercise can improve sleep quality and further promote well-being. Studies also found that sleep quality could significantly mediate the influences of predictors (e.g., perceived stress and physical exercise, excessive mobile phone use) of social adaptation (e.g., self-rated health and mental health) [31,32]. Therefore, we hypothesized that sleep quality could mediate the association between physical exercise and well-being.

Sleep quality is also closely associated with basic psychological need satisfaction [33]. Self-determination theory has been adopted as an integrated theoretical framework to explain the psychological predictors of sleep and its outcomes [18,34]. In particular, it points out that satisfying basic psychological needs are essential for good sleep quality and well-being, whereas frustration is deleterious, causing stress, negative affect, and sleep problems, which can damage individuals’ well-being [33,35]. Thus, from the perspective of self-determination theory, it was further hypothesized that physical exercise would be positively associated with well-being through the sequential mediating effect of basic psychological need satisfaction and sleep quality.

In summary, based on the daily life and social focus of undergraduate students, utilizing the perspective of self-determination theory, our study aimed to examine the association between physical exercise and undergraduate students’ subjective well-being, as well as the underlying mechanism—the mediating roles of basic psychological need satisfaction and sleep quality. Though the relations between the main variables have been examined independently, this study aimed to examine these relations comprehensively and uncover the underlying mechanism, which could significantly contribute to various aspects of the research in this field.

## 2. Materials and Methods

### 2.1. Participants

We recruited participants from a public university in China through convenience sampling (one of the most common methods of sampling in the social sciences). A total of 782 undergraduate students were recruited to participate in this study voluntarily, and 770 undergraduate students (464 male) aged between 18 and 22 years (mean age = 19.90 ± 1.15 years) finally completed the survey. The other 12 participants were excluded because of careless or missing answers to over 30% of the survey items.

### 2.2. Measurement

Physical exercise. We adopted a physical exercise questionnaire for undergraduate students to assess the participants’ exercise habits in daily life. The tool is widely used among Chinese undergraduate students [36]. Participants were asked to respond to each of the eight items (e.g., “I often take part in sports activities”) on a five-point scale (ranging from 1 = strongly disagree to 5 = strongly agree), with higher scores indicating a better physical exercise habit. In this study, the Cronbach’s alpha for the scale was 0.91.

Basic psychological need satisfaction in exercise. We adopted the Chinese version [37] of the Psychological Need Satisfaction in Exercise Scale [38] to assess the degree to which psychological needs could be satisfied by physical exercise. It consists of 15 items on the three dimensions of basic psychological needs: competence (five items, e.g., “I feel good about my ability in exercise”), autonomy (five items, e.g., “I am free to exercise in my own way”), and relatedness (five items, e.g., “I share a common bond with people through exercise”). Participants were asked to respond to each item on a seven-point scale (ranging from 1 = strongly disagree to 7 = strongly agree), with higher scores indicating a high degree of satisfaction of basic needs. In our study, the Cronbach’s alpha of this scale was 0.93.

Sleep quality. We used the Chinese version [39] of the Pittsburg Sleep Quality Index to measure sleep quality. This 18-item tool was developed to assess seven aspects of sleep quality (e.g., sleep latency, sleep durability, and sleep disturbances), with higher scores indicating worse sleep quality. The Cronbach’s alpha for this scale was 0.80 in our study.

Subjective well-being. Subjective well-being consists of two components: emotional or affective and judgmental or cognitive [40]. Accordingly, we adopted the Satisfaction with Life Scale (SWLS) [41] and the Chinese version of the Positive and Negative Affect Schedule (PANS) [42] to measure these two components [41]. The SWLS consists of five items rated on a seven-point scale (ranging from 1 = strongly disagree to 5 = strongly agree). The PANS measures positive affect (PA, nine items) and negative affect (NA, nine items) on a five-point Likert scale (ranging from 1 = very slightly to 4 = very strongly). Scores are normalized to generate a total score (by summing the standardized SWLS and PA scores and subtracting standardized NA scores), with higher scores indicating higher levels of subjective well-being [43]. In this study, the Cronbach’s alpha values of the SWLS and the PA and NA dimensions of the PANS were 0.92, 0.86, and 0.88, respectively.

### 2.3. Ethics Approval

This study was approved by the ethics committee for scientific research at the researchers’ affiliated institutions—Xi’an Jiaotong University. Our study adhered to the ethical values required for research with human beings, the fundamental principles included in the Helsinki Declaration (e.g., informed consent, protection of personal data, and guarantees of confidentiality), and the regulations of the education management department. Prior to participation in the study, all participants were informed of the principles of the study, and their written consent was obtained.

### 2.4. Statistical Analysis

All statistical analyses were conducted using IBM SPSS Statistics for Windows, Version 25.0, and Mplus 8.0. First, we conducted a confirmatory factor analysis to test for a significant common method bias. Second, we derived the descriptive statistics for the main research variables and then conducted Pearson’s correlation analysis to analyze the relations between them. Finally, we used the PROCESS macro (http://www.afhayes.com, accessed on 26 May 2022) for SPSS (Model 6, which was created to test a complex mediating model with multiple mediating roles) created by Hayes [44] to test the mediating model, with gender and age included as controls; it can not only report traditional regression results but also test the mediating effect directly with the bootstrap method. Specifically, a total of 1000 bias-corrected samples were set in this analysis, and the effect was considered to be significant if the 95% confidence interval did not contain zeroes.

## 3. Results

### 3.1. Test for Common Method Bias

The data in our study were collected through self-report questionnaires, which could have given rise to a common method bias. We adopted techniques (e.g., anonymity and reverse scoring) to alleviate this bias, following relevant suggestions. Moreover, to ensure the reliability and accuracy of the results, we conducted a confirmatory factor analysis to test the hypothesis that a single factor could account for all of the variances in the data [45]. The results revealed a poor model fit (χ^2^/df = 11.72, RMSEA = 0.31, TLI = 0.51, CFI = 0.55), indicating that the estimations of the relations among the variables had no significant biasing effects.

### 3.2. Descriptive Statistics and Correlations between Main Study Variables

Table 1 shows the mean and standard deviation for the main variables, as well as the correlation values. The main variables were significantly correlated. Physical exercise was positively associated with basic psychological need satisfaction (BPNS) in exercise and subjective well-being. All three variables were negatively correlated with sleep quality. BPNS in exercise was also positively correlated with subjective well-being.

### 3.3. Mediating Model Analyses

The results of the regression analysis for the mediating model are presented in Table 2, in which gender and age are included as control variables. Physical exercise was significantly associated with BPNS in exercise (β = 0.51, *p* < 0.001); and both physical exercise (β = −0.22, *p* < 0.001) and BPNS in exercise (β = −0.17, *p* < 0.001) were significantly associated with sleep quality. When these variables were entered into the regression model to predict subjective well-being, physical exercise (β = 0.20, *p* < 0.001), BPNS in exercise (β = 0.31, *p* < 0.001), and sleep quality (β = −0.23, *p* < 0.001) showed significant association with subjective well-being. Thus, BPNS in exercise and sleep quality partially mediated the association between physical exercise and subjective well-being.

The test on the mediating model, shown in Table 3, further showed that the mediating effect of BPNS in exercise and sleep quality in the association between physical exercise and subjective well-being was significant (total indirect effect = 0.25, 95% CI [0.19, 0.32]). The indirect effect contained three significant mediating pathways: physical exercise → BPNS in exercise → subjective well-being (indirect effect = 0.17, 95% CI [0.12, 0.23]); physical exercise → sleep quality → subjective well-being (indirect effect = 0.06, 95% CI [0.03, 0.09]); and physical exercise → BPNS in exercise → sleep quality→ subjective well-being (indirect effect = 0.03, 95% CI [0.01, 0.06]).

## 4. Discussion

Based on relevant research and the daily life of undergraduate students, this study examined the mediating mechanism underlying the association between physical exercise and subjective well-being and the roles of basic psychological need satisfaction and sleep quality from the perspective of self-determination theory. The results showed that physical exercise was positively associated with subjective well-being through the mediating roles of psychological need satisfaction and sleep quality.

Consistent with numerous studies on the benefits of physical exercise [2,11], our findings indicated that physical exercise was positively associated with undergraduate students’ adaptation and subjective well-being. It is well-established that regular physical exercise and exercise habits are indispensable components of a healthy lifestyle that bring great benefits to physical and psychological health and adaptation [9,12,42]. Particularly, physical exercise also contributes to people’s psychological health and social adaptation [2], and the good physical condition brought about by exercise also contributes directly to social adaptation. Physical exercise is also a positive way to cope with stress and negative life events, showing a buffering effect and decreasing perceived stress [17,46]. Relevant studies have also provided evidence from different perspectives: physical exercise is negatively related to the negative indicators of social adaptation (e.g., depression, anxiety, and suicide) [1,2,47] and positively related to positive indicators (e.g., positive self-conception, psychological capital, and good quality of life) [2,11,48]. From the opposite perspective, a lack of necessary physical exercise, such as sedentary behavior and excessive screen time, negatively influences individuals’ health and adaptation, being linked to obesity and overweight, depression, low quality of life, and low self-esteem [12,14]. Practically, physical exercise interventions have also been widely adopted to improve individuals’ physical and psychological functions (such as promoting sleep quality and positive emotions and decreasing perceived stress and anxiety) [15,16]. Nowadays, physical exercise may also be adopted to improve health conditions in stressful environments, such as the COVID-19 pandemic [10,17]. Our results confirmed that physical exercise, a key element of social adaptation, was positively associated with undergraduate students’ subjective well-being. We further examined the mediating mechanism underlying this relation and found that physical exercise was positively associated with undergraduate students’ subjective well-being through the independent and sequential mediating effects of sleep quality and basic psychological need satisfaction.

### 4.1. The Mediating Roles of Sleep Quality and Basic Psychological Need Satisfaction

First, sleep quality is not only an important indicator but also a key factor influencing physical and psychological health [49]. In addition to affecting work or learning efficiency, poor sleep quality can reduce the quality of daily life, particularly psychological distress and pain, resulting in diverse detrimental effects on people’s health and wellbeing [27,28,29]. Physical exercise is helpful in improving, and is closely associated with, sleep quality [12,31]. It has also been widely adopted as an intervention or therapy for poor or disordered sleep and has been found to have a positive effect on the improvement of sleep quality [29,50]. Thus, physical exercise can improve sleep quality and further increase subjective well-being. Our findings support previous studies that have shown the importance of good sleep quality and how it mediates the positive effects of physical exercise on individuals, which further confirms the importance of sleep quality in the health and well-being of undergraduates.

Secondly, regarding the mediating role of basic psychological need satisfaction, our findings fit well with the main points of self-determination theory: the satisfaction of basic psychological needs is one of the key factors accounting for good social adaptation [18,19,20]. In particular, the satisfaction of basic psychological needs fosters positive evaluation and affect [18,21], which is what subjective well-being is supposed to be, indicating its close association with subjective well-being. Physical exercise also contributes to the satisfaction of the three basic psychological needs [15,23,24]. For competence, physical exercise provides many opportunities to experience success or positive feedback in challenging physical tasks; for autonomy, when and how to exercise is largely dependent on the individual’s intention and interest; for relatedness, exercise may also encourage meaningful connection with others through similar interests and shared activities. Frequent exercisers also tend to have intrinsic motivation and are more likely to experience feelings of enjoyment in the exercise of their skills, personal accomplishment, and excitement [24,51].

Finally, the points of both self-determination theory and relevant empirical evidence indicate that basic psychological need satisfaction could improve sleep quality [34,35]. Combined with the positive role of sleep quality in well-being, physical exercise could boost psychological need satisfaction and further improve sleep quality, both of which contribute to higher levels of subjective well-being. Our findings likewise showed that physical exercise was positively associated with undergraduate students’ subjective well-being through the independent mediating effect of basic psychological need satisfaction and the sequential mediating effect of basic psychological need satisfaction and sleep quality. Thus, the satisfaction of basic psychological needs is essential for positive outcomes in physical activity contexts. It also inspires intrinsic motivation and stimulates individuals to engage in more activities [15,18], potentially initiating a virtuous circle for improving individuals’ health and well-being.

### 4.2. Limitations of the Study

The limitations of this study should be acknowledged. First, physical exercise was measured through self-reports, but differences are known to exist between objective and subjective measurements of physical exercise [52]. Future studies should adopt more objective measures; for example, smart wearable devices (such as smartwatches) could be used to measure physical activity in a more rigorous and objective way. Second, we focused on the frequency and positive outcomes of physical exercise, whereas excessive physical exercise may lead to deleterious outcomes, and the intensity and duration may have different influences [53,54]. Future studies should examine the influence of physical exercise from a more holistic perspective. Third, causal hypotheses could not be tested owing to our use of a cross-sectional design. Future work should include longitudinal, experimental, or intervention research methods to determine the causal nature of the relations. Finally, other mechanisms should be examined to clarify the relation between physical exercise and well-being.

## 5. Conclusions

Our study found a positive association between physical exercise and undergraduates’ subjective well-being. Psychological need satisfaction and sleep quality could significantly mediate this relation through three pathways: the independent mediating effects of basic psychological need satisfaction and sleep quality and the sequential mediating effect of basic psychological need satisfaction and sleep quality. These findings have both theoretical and practical implications.

Theoretically, this study expanded the research on the positive outcomes of physical exercise and the factors influencing subjective well-being. In particular, it reveals the mediating mechanism underlying the relation between physical exercise and subjective well-being by integrating the psychological and physical factors together. Based on previous studies, the findings further elucidate the positive influences of regular physical exercise and expand self-determination theory—it could be adopted to explain the physical exercise-related issues. Practically, individuals should acknowledge the benefits of physical exercise and participate in exercise rationally and frequently. Considering the important role of basic psychological need satisfaction, undergraduates should be guided to satisfy their basic psychological needs in a healthy way or through reasonable means (e.g., physical exercise). Finally, measures should also be adopted to encourage undergraduate students to engage in physical exercise to improve their sleep quality and subjective well-being.

## Figures and Tables

**Table 1:** Descriptive statistics and correlations among the main variables.

Variables	*M*	*SD*	1	2	3	4
1. Physical exercise	3.21	0.91	1			
2. BPNS in exercise	3.68	0.76	0.58 ***	1		
3. Sleep quality	6.25	3.27	−0.34 ***	−0.33 ***	1	
4. Subjective well-being	0	1.70	0.43 **	0.48 ***	−0.38 ***	1

Note: BPNS in exercise = basic psychological need satisfaction; ** *p* < 0.01, *** *p* < 0.001.

**Table 2:** Regression models analysis.

Dependent Variables	Independent Variables	*R* ^2^	*F*	*β*	*t*	95% CI
BPNS in exercise	Gender	0.31	60.16 ***	0.03	0.86	[−0.06, 0.07]
	Age			−0.04	−1.36	[−1.02, 0.02]
	Physical exercise			0.51	17.86 ***	[0.46, 0.58]
Sleep quality	Gender	0.15	25.08 ***	−0.02	−0.49	[−0.09, 0.05]
	Age			−0.05	−1.38	[−0.11, −0.09]
	Physical exercise			−0.22	−5.12 ***	[−0.31, −0.14]
	BPNS in exercise			−0.17	−4.11 ***	[−0.26, −0.09]
Subjective well-being	Gender	0.37	90.18 ***	0.04	1.12	[−0.04, 0.13]
	Age			−0.07	−2.18 *	[−0.13, −0.01]
	Physical exercise			0.20	4.80 ***	[0.12, 0.28]
	BPNS in exercise			0.31	7.51 ***	[0.23, 0.40]
	Sleep quality			−0.23	−6.11 ***	[−0.29, −0.15]

Note: Gender: 0 = female, 1 = male; * *p* < 0.05, *** *p* < 0.001.

**Table 3:** Test for mediating effects.

	Standardized Value	Bootstrap LLCI	Bootstrap ULCI
Total mediating effect	0.25	0.19	0.32
Physical exercise → BPNS in exercise → subjective well-being	0.17	0.12	0.23
Physical exercise → sleep quality → subjective well-being	0.06	0.03	0.09
Physical exercise → BPNS in exercise → sleep quality→ subjective well-being	0.03	0.01	0.06

## Data Availability

The data from this study are available from the corresponding author upon reasonable request.
